# New light into the hormogastrid riddle: morphological and molecular description of *Hormogaster joseantonioi* sp. n. (Annelida, Clitellata, Hormogastridae)

**DOI:** 10.3897/zookeys.414.7665

**Published:** 2014-06-05

**Authors:** Daniel Fernández Marchán, Rosa Fernández, Marta Novo, Darío J. Díaz Cosín

**Affiliations:** 1 Departamento de Zoología y Antropología Física, Facultad de Biología, Universidad Complutense de Madrid, C/ José Antonio Nováis 2, 28040, Madrid, Spain; 2 Museum of Comparative Zoology, Department of Organismic and Evolutionary Biology, Harvard University, 26 Oxford Street, Cambridge, MA 02138, USA; 3 Cardiff School of Biosciences, Cardiff University, BIOSI 1, Museum Avenue, Cardiff CF10 3AT, UK

**Keywords:** Species description, earthworm, integrative taxonomy, phylogeny, disjunct distribution

## Abstract

The earthworm family Hormogastridae shows a remarkable disjunction in its distribution in the Iberian Peninsula, with the *Hormogaster elisae* species complex isolated from the rest of the species. *Hormogaster joseantonioi* **sp. n.**, a new species found in the intermediate area between the main ranges (in Teruel, Aragón), was described following the integrative approach, as it is suitable for earthworms due to their highly homoplasic morphology. The phylogenetic analysis of the molecular markers placed the new species as a sister taxon to *H. elisae*, thus showing the colonizing lineage of Central Iberian Peninsula could have originated near the *H. joseantonioi* **sp. n.** current range. External morphological characters revealed some degree of overlap with previously described species, but internal characters presented configurations/states unknown from other members of the family. These traits make the new species a key piece to understand the evolution of Hormogastridae.

## Introduction

The increasing availability of molecular and ecological data has placed the integrative taxonomy (as defined by Dayrat 2005) as a viable alternative to traditional species description. Several authors advocate its use in different animal groups (Padial and De La Riba 2010; Schlick-Steiner et al. 2010; Heethoff et al. 2011; but see Yeates et al. 2011 for iterative taxonomy instead) and particularly in earthworms (Blakemore and Kupriyanova 2010; Novo et al. 2012), whose taxonomy is in need of deep revision in the light of molecular phylogeny (Jamieson et al. 2002; Pop et al. 2003, 2007; Chang et al. 2008; Briones et al. 2009; Pérez-Losada et al. 2009, 2011; Novo et al. 2011; Fernández et al. 2012).

Fernández et al. (2014) have developed a new tool based in micro-computed tomography to study specimens in a non-destructive way which could help as an additional source of information.

Taxonomic characters traditionally used for the study of earthworms are few and sometimes present high intraspecific variability (Michaelsen 1900 and Stephenson 1930 on their global fauna; Pop et al. 2003 and Briones et al. 2009 about lumbricid earthworms). Recent findings show that cryptic diversity is common in these animals (but see critique in Blakemore et al. 2010), therefore earthworm taxonomy can particularly benefit from an integrative approach.

Novo et al. (2011) presented a molecular phylogeny of Hormogastridae (Oligochaeta, Annelida), whose taxonomy has historically been built on morphological characters, which highlighted some interesting evolutionary aspects. On one hand, hormogastrid distribution across the Western Mediterranean is biogeographically consistent, reflecting the geological events that affected the region in the Tertiary (which confirms previous studies, e.g Bouché 1972, Sbordoni et al. 1992). Two species -*Xana omodeoi* Diaz Cosin, Briones & Trigo, 1989 and the morphospecies *Hormogaster elisae* Álvarez, 1977 -, however, are found in locations far apart from the family main range in the Iberian Peninsula. While all the other Iberian species are distributed in Northeastern Spain, *Xana omodeoi* inhabits Northwestern Spain and *Hormogaster elisae* is found in Central Spain (Segovia, Madrid and Guadalajara). The result is a disjunct distribution.

Novo (2010) found *Hormogaster elisae* complex to be monophyletic, and thus the likely result of a single colonisation event presumably from the North or the East of the Iberian Peninsula. There could be remaining populations of the migrating lineage in the geographic gap, which haven’t been discovered yet.

On the other hand, it seems that most key characters used for hormogastrid traditional taxonomy and phylogeny (notably the shape, number and position of the spermathecae) are highly homoplasic, showing little or no phylogenetic signal across the family.

Due to its relevance for this subject, the intermediate area between the main ranges of hormogastrids in Spain has been subject to recent sampling campaigns. Both Zaragoza and Teruel (Aragón, Spain) were suitable regions as they have been poorly sampled for earthworms unlike the surrounding provinces. While no success was met in Zaragoza, a population assignable to a new species of Hormogastridae was recently found in Teruel.

This paper focuses on the description of *Hormogaster joseantonioi* sp. n. from an integrative point of view, following the example of Novo et al. (2012). The new molecular and morphological data are interpreted to gain insight into the diversification and morphological radiation of the family, with some considerations about its constituent genera.

## Materials and methods

### Earthworm specimens and sampling points

Specimens were collected by hand and fixed in the field in ca. 96% EtOH, with subsequent alcohol changes. Once in the laboratory, specimens were preserved at -20 °C.

**Figure 1.**
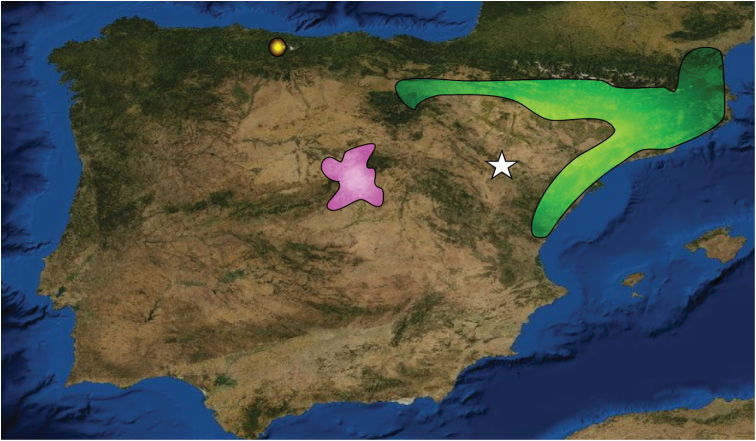
Map of the Iberian Peninsula showing the collection site of *Hormogaster joseantonioi* sp.n. (indicated by the white star). The northeastern hormogastrid range is shown in green, *Hormogaster elisae* range is shown in pink and *Xana omodeoi* known location is indicated in yellow.

The studied material includes 10 specimens (five mature specimens, one semimature specimen with tubercula pubertatis and four immatures) collected in a cleared holm-oak wood at the foothill of Sierra de Oriche, road A-2514 between Huesa del Común and Rudilla, Teruel (Spain) (41°0'55.68"N, 0°58'55.98"W) (Figure 1).

Specimens have been deposited in the Oligochaete collection of the Departamento de Zoología y Antropología Física, Universidad Complutense de Madrid (UCMLT), Spain with vouchers UCMLT 00001-00010.

Specimens available from previous studies (Novo et al. 2010, 2011, 2012) of all known hormogastrid species were used for comparison. Morphological characters include those features traditionally used for hormogastrids and other earthworms.

### Molecular data generation

Total genomic DNA was extracted from ventral integument tissue samples using the DNeasy Tissue Kit (QIAGEN) with two consecutive steps of elution (70 µl of buffer). Seven molecular regions were amplified: mitochondrial subunit 1 of cytochrome *c* oxidase (COI), 16S rRNA and tRNA Leu, Ala, and Ser (16S t-RNAs), one nuclear ribosomal gene (a fragment of 28S rRNA) and one nuclear protein-encoding gene (histone H3). Primer sequences, polymerase chain reactions (PCR) and sequencing reactions are the same as in Novo et al. (2011). GeneBank accession numbers for the holo- and paragenetypes, following Chakrabarty (2010) for the markers analysed here are shown in Table 1.

**Table 1.** Holo- and paragenetypes (sensu Chakrabarty 2010) of *Hormogaster joseantonioi* sp. n., and their GenBank accession numbers. The hologenetype is shown in bold.

Specimen	Voucher	COI	16S-tRNAs	28S rRNA	H3
HRUD1	UCMLT 00001	KJ632674	KJ632684	KJ632686	KJ632688
HRUD2	UCMLT 00002	KJ632675	KJ632685	KJ632687	KJ632689
**HRUD3**	**UCMLT 00003**	**KJ632676**			
HRUD4	UCMLT 00004	KJ632677			
HRUD5	UCMLT 00005	KJ632678			
HRUD6	UCMLT 00006	KJ632679			
HRUD7	UCMLT 00007	KJ632680			
HRUD8	UCMLT 00008	KJ632681			
HRUD9	UCMLT 00009	KJ632682			
HRUD10	UCMLT 00010	KJ632683			

### Phylogenetic analyses

The new sequences were combined with all the hormogastrid information available from previous studies (Novo et al. 2010, 2011, 2012) in order to find their phylogenetic placement inside the family. *Pontodrilus litoralis* Grube, 1855, *Dichogaster saliens* Beddard, 1893, *Amynthas robustus* Perrier, 1872, *Lumbricus terrestris* Linnaeus, 1758 and *Aporrectodea trapezoides* Dugès, 1828 were used as outgroups (all the GenBank accession numbers are shown in Appendix). As hormogastrid individuals from the same locality usually cluster together, one individual was analysed as representative per sampling site.

Sequences of each individual gene were aligned in MAFFT (Katoh and Standley 2013) with default settings and concatenated, resulting in a matrix of 2532 bp. jModelTest v. 2.1.3 (Darriba et al. 2012) was used to select the best-fit evolutionary model using the Akaike information criterion (AIC; Akaike 1973), and Bayesian information criterion (BIC; Schwarz 1978) which were GTR+I+G for COI, 16s and 28s, and HKY+I+G for H3.

Bayesian Inference (BI) of the phylogeny was estimated with MRBAYES v.3.1.2 (Ronquist and Huelsenbeck 2003) implemented in the CIPRES Science Gateway V. 3.3. (http://www.phylo.org/index.php/portal/). Unlinked nucleotide substitution models selected were specified for each gene fragment and the nucleotide substitution estimates were allowed to vary independently between each partition. Parameters were set to ten million generations and 10,000 trees were sampled for every 1000th generation, initiating the analysis from a random tree. After two analysis were performed 20% of the trees were discarded as burn-in, and the remaining trees were combined to find the maximum a posteriori probability estimate of phylogeny. Maximum likelihood analyses were performed with RAxML 7.2.7 (Stamatakis 2006) in the CIPRES Science Gateway with default settings, using GTR+I+G for each data partition and estimating the support for the resulting topologies by 100 bootstrap replicates.

Uncorrected pairwise differences for the mitochondrial regions were calculated between *Hormogaster joseantonioi* and the most closely related species with Arlequin 3.5 (Excoffier and Lischer 2010. To visualize the genetic distance we constructed networks with SplitsTree4 v.4.11.3 (Huson and Bryant 2006) for the more variable genes, including the former species plus *Hormogaster riojana* Qiu & Bouché, 1998 and *Aporrectodea trapezoides* as outgroups. Default settings were used.

## Results

### Taxonomic results
Phylum Annelida Lamarck, 1802
Subphylum Clitellata Michaelsen, 1919
Class Oligochaeta Grube, 1850
Superorder Megadrilacea Benham, 1890
Order Haplotaxida Michaelsen, 1900
Family Hormogastridae Michaelsen, 1900
Genus *Hormogaster* Rosa, 1887

**Type-species.** *Hormogaster redii* Rosa, 1887.

#### Hormogaster joseantonioi

Fernández Marchán sp. n.

http://zoobank.org/1B7B13C0-FA56-466E-9FFE-AB985EB582BA

http://species-id.net/wiki/Hormogaster_joseantonioi

##### Material examined.

Holotype. Adult (UCMLT 00003), 41°0'55.68"N, 0°58'55.98"W, from a cleared holm-oak wood on the foothill of Oriche mountains, road A-2514 between Huesa del Común and Rudilla, Teruel (Spain), collectors D. Fernández Marchán and J.A. Fernández Fernández.

**Paratypes.** Nine individuals (UCMLT 00001, 00002, 00004-00010), with the same collection data of the holotype.

**Other material examined.** 16 hormogastrid species and several subspecies belonging to the UCMLT collection.

##### Morphological description.

**Figure 2.**
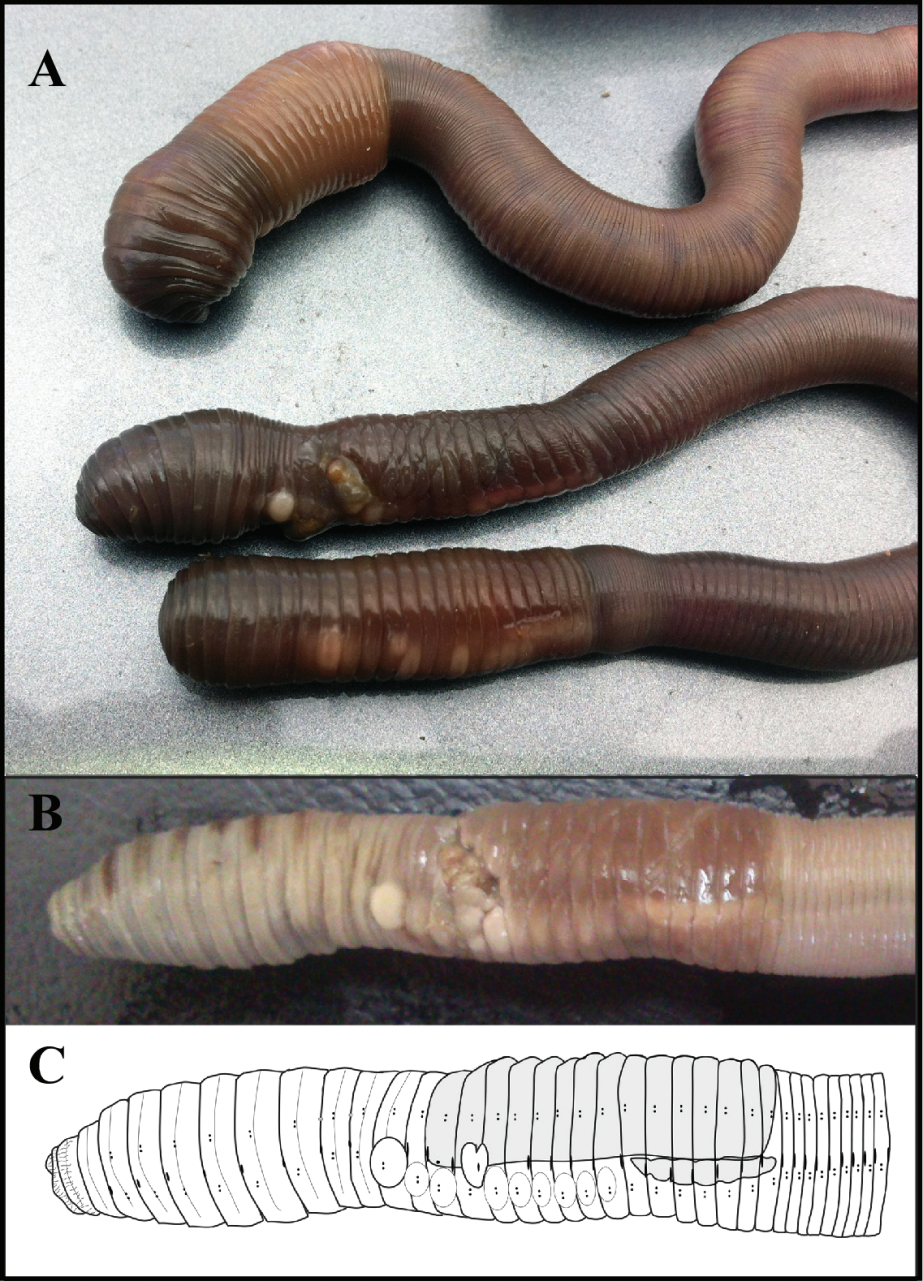
(**A)** Live specimens of *Hormogaster joseantonioi* sp.n. External morphology of a fixed specimen, shown in a picture (**B**) and diagram (**C**).

*External morphology* (Figure 2). *Measures taken on the two only complete specimens, one being the holotype.

Length of mature specimens*: 178–180 mm.

Maximum diameter (pre-clitellar, clitellar, post-clitellar) of mature specimens: 8–10, 9–11, 7–10 mm.

Number of segments*: 305–369.

Weight (fixed specimens)*: 7.05–11.57 g.

Colour: From light brown to dark chocolate brown varying between individuals, with orangeish-brown clitellum of a lighter shade on living specimens (Figure 2a). Beige with brown stripes or patches, mainly on the anterior end, with darker clitellum on fixed specimens (Figure 2b).

Prostomium prolobic, longitudinal striation on segments 1 and 2.

Closely paired chaetae; interchaetal ratio at segment 40, *aa*: 33, *ab*: 1.3, *bc*: 6, *cd*: 1, *dd*: 27. Nephridial pores in a row between chaetae *b* and *c* (very close to *b*), visible on fixed specimens as a brownish line.

Spermathecal pores at intersegments 9/10 and 10/11 at the level of *cd*.

Male pores open over chaetae *ab* at the intersegment 15/16, surrounded by heart-shaped porophores which extend over most of segment 15 and at least half of 16. Female pores in segment 14 at the same level as male pores.

Clitellum saddle-shaped extending over segments (13) 14–28. Tubercula pubertatis on 1/n 22-27(1/n 28) as a continuous line. Papillae of chaetae ab in variable positions, usually between segments 12 and 28: papillae on 12 always showing an unusual degree of development in mature individuals, being very conspicuous both in live and fixed specimens (Figure 2a).

##### Internal anatomy.

Funnel shaped, strongly thickened septa in 6/7, 7/8 and 8/9, septum 9/10 slightly thickened. The latter’s attachment to the dorsal body wall is displaced two segments backwards, creating a mismatch between inner and outer segmentation with an internally very wide segment 9.

Last pair of hearts in segment 11. Three shiny, strongly muscular gizzards in 6, 7 and 8. Not apparent Morren’s glands, even though small wrinkles exist in the oesophageal wall between segments 10 and 16.

A posterior gizzard is not well differentiated. There is a slight dilatation of the oesophagus between 14 and 16, but it lacks the muscular wall and reinforcements of a true gizzard. First section of the intestine is not dilated.

Typhlosole begins around segments 20 and 21 with seven lamellae, which around segments 26–27 increase to nine. From there they decrease gradually in number until segments 80–105, where they fuse in a single lamella. The latter extends until segments 218-230, where the typhlosole ends.

Fraying testes and iridescent seminal funnels in 10 and 11. Two pairs of voluminous, grainy seminal vesicles in 11 and 12. Ovaries and female funnels in 13, ovisacs in 14.

**Figure 3.**
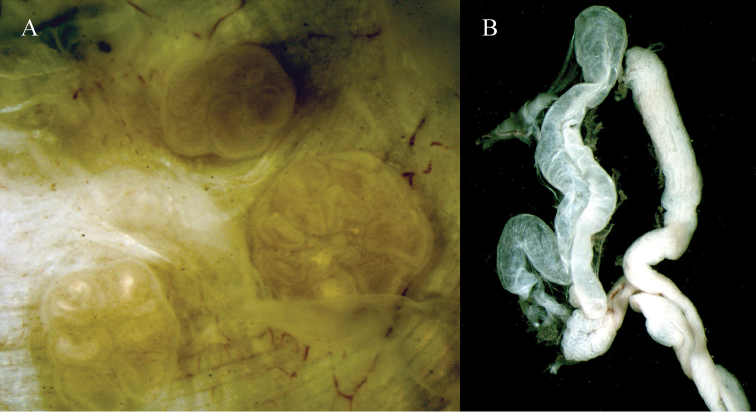
**A)** Spermathecae in segments 9 and 10. Note the double spermathecae in segment 10 of this specimen. **B**) Nephridial bladder of segment 7.

Two pairs of spermathecae in intersegments 9/10 and 10/11 (but apparently contained in segment 9 due to septum’s backward displacement), the posterior pair bigger. They are sessile and disc-shaped, with multiple inner chambers which open to the exterior through a common pore, in the intersegments 9/10 and 10/11. Some individuals show double spermathecae (each multicameral and with own pore), either in 9/10 or 10/11 (Figure 3a).

Anterior nephridial bladders U-shaped with very close branches and no apparent cecum (Figure 3b). Bladders gradually flatten towards the end of the body, taking the usual elongated shape.

##### Distribution.

Known only from its type locality.

##### Habitat.

The specimens were collected at 10–20 cm deep in the soil in a cleared holm-oak wood, at the border between a dense forest of *Quercus rotundifolia* and a dryland farm. The soil had the following characteristics: 23.03% coarse sand, 8.06% fine sand, 5.33% coarse silt, 60.74% fine silt, and 2.84% clay, constituting a silty loam soil, carbon (C): 2.40%, nitrogen (N): 0.24%, C/N: 10.18, pH: 7.98. Mean annual temperature is 12.7 °C and mean annual precipitation is 447.2 mm, as indicated by the nearest weather station (in Herrera de Los Navarros, Zaragoza-23 km away http://www.aragon.es/DepartamentosOrganismosPublicos/Organismos/InstitutoAragonesEstadistica/AreasTematicas/14_Medio_Ambiente_Y_Energia/ci.05_Clima_Datos_climatologicos.detalleDepartamento?channelSelected=ea9fa856c66de310VgnVCM2000002f551bacRCRD#section1).

##### Etymology.

The species is named after Jose Antonio Fernández Fernández, father of the first author Daniel Fernández Marchán and important contributor during the sampling campaign in which this species was discovered.

##### Molecular characters.

Analyses were conducted on sequences from loci COI (10 individuals), 16S (2 individuals), 28S (2 individuals) and H3 (2 individuals) of the new species, combined with similar sequences from other hormogastrid species.

**Figure 4.**
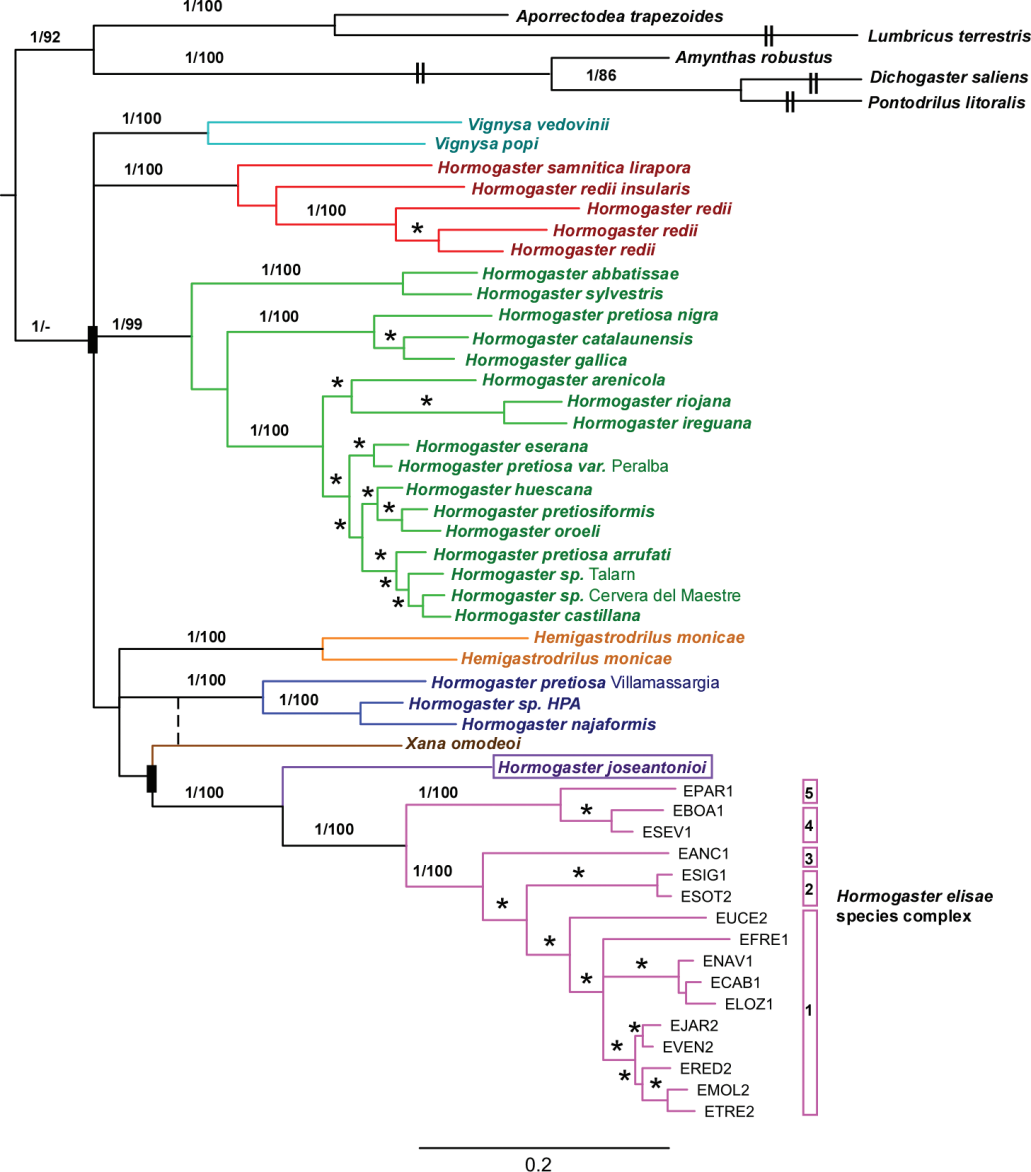
Bayesian inference of the phylogenetic tree on the concatenated sequence. Numbers above branches indicate posterior probability/bootstrap (of the Maximum Likelihood analysis) support values higher than 0.9/70 (shown as asterisks on terminal branches). Black rectangles show clades not recovered in both analyses (the alternative is shown with a dashed line). The cryptic species included in *Hormogaster elisae* are numbered from 1 to 5 (following Novo et al. 2010).

The resulting Bayesian inference of the phylogenetic tree is shown in Figure 4. Its topology was congruent with that of the Maximum Likelihood inferred tree, except for the different placement of *Xana omodeoi*. *Hormogaster joseantonioi* sp.n. was recovered as a monophyletic clade, with the *Hormogaster elisae* species complex as a sister clade.

Uncorrected pairwise distances for the genes COI and 16S-tRNA for *Hormogaster joseantonioi* and the species within the same clade (with *Hormogaster elisae* divided into its five cryptic species) are shown in Table 2.

**Table 2.** Uncorrected pairwise distances for the genes COI (below the diagonal) and 16S-tRNA (above the diagonal) for *Hormogaster joseantonioi* and the species on the same clade. XAN – *Xana omodeoi*, HPRE – *Hormogaster pretiosa*, HNAJ – *Hormogaster najaformis*, HEM – two populations of *Hemigastrodrilus monicae*. Intraspecific divergence for COI/16S is shown in the diagonal.

	HJOS	HE3	HE1	HE2	HE5	HE4	XAN	HPRE	HNAJ	HEM*	HEM**
**HJOS**	0.14/0	13.10	14.20	12.50	19.41	13.50	14.23	14.28	15.31	17.40	16.07
HE3	18.10	0.29/0	9.87	9.96	17.18	12.34	14.37	15.93	16.69	17.54	15.57
HE1	17.77	15.51	10.03/4.10	7.97	17.83	12.95	15.54	17.73	17.54	17.26	16.56
HE2	16.47	14.16	15.13	1.75/0.67	17.03	13.38	14.93	16.62	18.18	16.70	16.70
HE5	16.83	16.28	17.48	16.36	0.34/0	16.37	21.04	21.55	22.37	22.28	21.32
HE4	19.08	15.67	17.37	16.86	10.38	3.75/1.75	15.49	18.06	17.51	17.81	16.53
XAN	18.30	18.26	18.36	18.96	17.01	18.49	0.37/0.19	11.60	13.58	14.34	12.66
HPRE	18.61	20.17	20.34	19.74	18.92	19.52	17.76	0/2.14	10.74	16.47	13.69
HNAJ	18.92	18.39	19.77	18.19	18.64	19.17	19.92	17.31	0.10/0.18	16.69	14.86
HEM*	18.38	18.52	19.17	20.45	17.06	18.58	20.45	19.67	19.92	3.50/1.97	8.76
HEM**	18.11	18.19	18.10	17.79	16.14	16.55	18.31	19.24	18.93	17.63	6.30/2.07

## Discussion

Both morphological and molecular characters of *Hormogaster joseantonioi* sp.n. separate it clearly from all known hormogastrid species, the number of typhosole lamellae and the kind and location of the spermathecae being particularly distinctive. Those characters, while failing to resolve internal relationships within Hormogastridae, have been shown to be suitable for species diagnosis (Rota 1993 on typhlosole importance; Novo et al. 2012 on spermathecae number to separate *Hormogaster abbatissae* from *Hormogaster sylvestris*).

The species *Hormogaster riojana*, while distantly related according to molecular phylogeny, shows many similarities in morphology to *Hormogaster joseantonioi* (Table 3). However, *Hormogaster joseantonioi* differ by its lower number of lamellae in its typhlosole and shorter tubercula pubertatis. Moreover it is longer and heavier. While the two species share a very similar position and shape of the spermathecae, some *Hormogaster joseantonioi* individuals show an additional spermatheca in segment 10 (on the right or left side). These cases don’t seem to be teratologic, as the supernumerary spermathecae have their own pore in the body surface and contain sperm, thus being fully functional.

**Table 3.** Comparison of the morphological characters of *Hormogaster joseantonioi* sp. n. and some of the phylogenetically closest species (*Hormogaster elisae*, *Xana omodeoi* and *Hormogaster najaformis* Qiu & Bouché, 1998) plus the distantly related *Hormogaster riojana* and *Hormogaster castillana* Qiu & Bouché, 1998. N. segments: number of segments. N. typhlosole lamellae: number of typhlosole lamellae. Body length, weight and number of segments refer to adult specimens.

	*Hormogaster joseantonioi*	*Hormogaster elisae*	*Xana omodeoi*	*Hormogaster najaformis*	*Hormogaster riojana*	*Hormogaster castillana*
Colour	Brownish	Colourless	Colourless	Slightly greyish	Dark brownish	Brownish grey
Clitellum	(13)14–28	(13)14(15)–26(27)28	14–26	13–31	13,14,17–27,28	1/14,15–29,1/2 30
Tubercula pubertatis	1/n 22–27 (1/n 28)	22(23)–25(26)	23–26	20–26	(20)21–27	22–28
Length (mm)	178–180	92–200	20–161	188–230	154	200–325
N. segments	305–369	205–300	190–230	395–523	243–278	320–429
Weight (g)	7.05–11.57	1.96–9.67	0.59–4.23	22.6–31.4	6.57	12.85–29.38
Spermathecae position (pores) and appearance	9 (see text) (9/10,10/11) Simple(double) Multicameral, disc shaped	9,10 (9/10,10/11) Simple Tubular	10,11 (9/10,10/11) Simple Small, globular	10,11 (10/11,11/12) Multiple Small, globular	9,10 (9/10,10/11) Simple Multicameral, disc shaped	9,10 (9/10,10/11) Simple Globular
N. typhlosole lamellae	9	5	12	15–17	15	21–23
Thickened septa	6/7,7/8,8/9, (9/10)	6/7,7/8,8/9, (9/10)	(6/7),7/8,8/9, 9/10,(10/11)	6/7,7/8,8/9, (9/10)	7/8,8/9,9/10, (10/11)	7/8,8/9,9/10, (10/11)

Other hormogastrid species possess double or multiple spermathecae, but never of the multicameral, disc shaped kind.

The geographically closest species, *Hormogaster castillana* (from Puerto Querol, Castellón), is neither morphologically nor phylogenetically closely related (Table 3).

*Hormogaster joseantonioi* sp. n. appears nested on a weakly supported clade on the phylogenetic tree, consisting in *Hemigastrodrilus monicae*, *Xana omodeoi*, *Hormogaster pretiosa* from Villamassargia, *Hormogaster najaformis* (and HPA from Omodeo, see Novo et al. 2011) and *Hormogaster elisae*. Genetic distances were high in all cases (16.47–19.08% for COI, 12.50–17.40% for 16S) according to the reference intervals given by Chang and James (2011). Aside from *Hormogaster elisae*, none of them showed significant morphological likeness to the new species, with the very different spermathecae configurations being noteworthy (Table 3).

The *Hormogaster elisae* morphospecies was recovered as sister clade to *Hormogaster joseantonioi* sp. n. with high support. From a morphological point of view, most of their external characters overlap, except for a slightly longer clitellum and tubercula pubertatis, bigger average size and stronger pigmentation in *Hormogaster joseantonioi* sp. n. However, internal characters are very different and these species match neither in the number of lamellae in the typhlosole (five versus nine) nor in the structure of the spermathecae, which are tubular in *Hormogaster elisae* and disc-shaped and multicameral in *Hormogaster joseantonioi*. It’s worth noting that *Hormogaster elisae* shares the backwardly displaced disposition of the 9/10 septum.

Based on their phylogenetic and morphological relatedness, an origin of *Hormogaster elisae* from a common ancestor with *Hormogaster joseantonioi* sp. n. seems likely. This scenario is sensible from a biogeographical point of view, as the locality of the new species is intermediate between the ranges of *Hormogaster elisae* and the northeastern main hormogastrid range. A connection of emerged lands would have been possible from the Cretaceous-Tertiary boundary onwards (Andeweg 2002).

While *Hormogaster joseantonioi* status as a good species and its phylogenetic relationships seem quite clear, generic assignment is a more problematic matter. Novo et al. (2011) recovered the genus *Hormogaster* as paraphyletic in their molecular phylogeny, pointing out the need for a deep taxonomical revision of the family Hormogastridae, currently in preparation (author’s work in progress).

Based on its distinctive morphology and geographic range, high genetic divergence and consistent recovery as a well-defined clade, Novo (2010) suggested the *Hormogaster elisae* species complex should be established as an independent genus. Due to the close phylogenetic position and morphological similarity of *Hormogaster joseantonioi* to this clade it could be argued they both should be included in the same genus.

At this stage it is more conservative to assign *Hormogaster joseantonioi* to the genus *Hormogaster* until the revision of the family is completed, which will allow to establish (if possible) a well-founded genera system on Hormogastridae. This work narrows the discontinuity between the North-Eastern and Central ranges of the Spanish hormogastrids. At the same time it highlights the importance of an intensive sampling of the area between Teruel and the center of the Iberian Peninsula (mainly zones of Soria and Guadalajara) to hopefully find new species along the hypothetical colonization route.

## Supplementary Material

XML Treatment for Hormogaster joseantonioi

## Appendix

**Supplementary material.** GenBank accession numbers for all sequences used in the phylogenetic analysis, including outgroups. RF: sequences provided by Rosa Fernández.

Species	COI	16S-tRNAs	28S-rRNA	H3
*Hormogaster castillana* QUE	HQ621989	HQ621883	HQ621960.1	HQ622028
*Hormogaster elisae* 3 ANC	EF653870	GQ409754.1	GQ409657.1	HQ622001
*Hormogaster elisae* 4 BOA	GQ409661.1	GQ409704.1	GQ409656.1	HQ622004
*Hormogaster elisae* 1 CAB	GQ409689.1	GQ409729.1	GQ409653.1	HQ622007
*Hormogaster elisae* 1 FRE	GQ409698.1	GQ409723.1	GQ409653.1	HQ622009
*Hormogaster elisae* 1 JAR	GQ409665.1	GQ409745.1	GQ409653.1	HQ622013
*Hormogaster elisae* 1 LOZ	EF653888	GQ409725.1	GQ409653.1	HQ622016
*Hormogaster elisae* 1 MOL	EF653875	GQ409732.1	GQ409653.1	HQ622019
*Hormogaster elisae* 1 NAV	GQ409683.1	GQ409730.1	GQ409653.1	HQ622021
*Hormogaster elisae* 5 PAR	EF653898	GQ409709.1	GQ409655.1	HQ622024
*Hormogaster elisae* 1 RED	EF653881	GQ409741.1	GQ409653.1	HQ622029
*Hormogaster elisae* 4 SEV	EF653905	GQ409707.1	GQ409656.1	HQ622031
*Hormogaster elisae* 2 SIG	EF653893	GQ409710.1	GQ409654.1	HQ622033
*Hormogaster elisae* 2 SOT	GQ409700.1	GQ409716.1	GQ409654.1	HQ622034
*Hormogaster elisae* 1 TRE	GQ409678.1	GQ409737.1	GQ409653.1	HQ622038
*Hormogaster elisae* 1 UCE	GQ409692.1	GQ409720.1	GQ409653.1	HQ622039
*Hormogaster elisae* 1 VEN	GQ409671.1	GQ409750.1	GQ409653.1	HQ622041
*Hormogaster pretiosa arrufati*	HQ621995	HQ621889	HQ621966.1	HQ622040
*Hormogaster pretiosa* var. PRB	HQ621987	HQ621881	HQ621958.1	HQ622026
*Hormogaster pretiosa* Villamassargia	HQ621998	HQ621893	HQ621969.1	HQ622045
*Hormogaster pretiosiformis oroeli*	HQ621984	HQ621877	HQ621955.1	HQ622022
*Hormogaster redii redii*	HQ621978	HQ621871	HQ621949.1	HQ622012
*Hormogaster redii redii*	HQ621971	HQ621863	HQ621942.1	HQ622000
*Hormogaster redii redii*	HQ621976	HQ621869	HQ621947.1	HQ622010
*Hormogaster redii insularis*	HQ621996	HQ621890	HQ621967.1	HQ622042
*Hormogaster samnitica lirapora*	HQ621993	HQ621887	HQ621964.1	HQ622036
*Hemigastrodrilus monicae*	HQ621979	HQ621872	HQ621950.1	HQ622014
*Hemigastrodrilus monicae*	HQ621982	HQ621875	HQ621953.1	HQ622018
*Hormogaster abbatissae*	HQ621990	HQ621884	HQ621961.1	HQ622030
*Hormogaster arenicola*	HQ621972	HQ621865	HQ621943.1	HQ622003
*Hormogaster catalaunensis*	HQ621973	HQ621866	HQ621944.1	HQ622005
*Hormogaster eserana*	HQ621977	HQ621870	HQ621948.1	HQ622011
*Hormogaster gallica*	HQ621974	HQ621867	HQ621945.1	HQ622006
*Hormogaster huescana*	HQ621980	HQ621873	HQ621951.1	HQ622015
*Hormogaster ireguana*	HQ621994	HQ621888	HQ621965.1	HQ622037
*Hormogaster najaformis*	HQ621985	HQ621878	HQ621956.1	HQ622023
*Hormogaster nigra*	HQ621988	HQ621882	HQ621959.1	HQ622027
*Hormogaster pretiosiformis*	HQ621983	HQ621876	HQ621954.1	HQ622020
*Hormogaster riojana*	HQ621970	HQ621862	HQ621941.1	HQ621999
*Hormogaster* sp. CER	HQ621975	HQ621868	HQ621946.1	HQ622008
*Hormogaster* sp. HPA	-	HQ621892	-	HQ622044
*Hormogaster* sp. TAL	HQ621992	HQ621886	HQ621963.1	HQ622035
*Hormogaster sylvestris*	HQ621981	HQ621874	HQ621952.1	HQ622017
*Vignysa popi*	HQ621991	HQ621885	HQ621962.1	HQ622032
*Vignysa vedovinii*	HQ621986	HQ621880	HQ621957.1	HQ622025
*Xana omodeoi*	HQ621997	HQ621891	HQ621968.1	HQ622043
*Amynthas robustus*	EF077569.1	EF490524.1	EF490529.1	-
*Dichogaster saliens*	-	AF406573.1	AY101560.1	-
*Pontodrilus litoralis*	-	AY340473.1	-	-
*Lumbricus terrestris*	HQ691222	U24570	HQ691218	HQ691227
*Aporrectodea trapezoides*	RF	HQ621864	RF	HQ622002
